# Large-Scale Indoor Visual–Geometric Multimodal Dataset and Benchmark for Novel View Synthesis

**DOI:** 10.3390/s24175798

**Published:** 2024-09-06

**Authors:** Junming Cao, Xiting Zhao, Sören Schwertfeger

**Affiliations:** 1 Shanghai Advanced Research Institute, Chinese Academy of Sciences, Shanghai 201210, China; caojm@sari.ac.cn; 2 University of Chinese Academy of Sciences, Beijing 100049, China; 3 Key Laboratory of Intelligent Perception and Human-Machine Collaboration, ShanghaiTech University, Ministry of Education, Shanghai 201210, China; zhaoxt@shanghaitech.edu.cn

**Keywords:** novel view synthesis, 3D reconstruction, indoor dataset, benchmark

## Abstract

The accurate reconstruction of indoor environments is crucial for applications in augmented reality, virtual reality, and robotics. However, existing indoor datasets are often limited in scale, lack ground truth point clouds, and provide insufficient viewpoints, which impedes the development of robust novel view synthesis (NVS) techniques. To address these limitations, we introduce a new large-scale indoor dataset that features diverse and challenging scenes, including basements and long corridors. This dataset offers panoramic image sequences for comprehensive coverage, high-resolution point clouds, meshes, and textures as ground truth, and a novel benchmark specifically designed to evaluate NVS algorithms in complex indoor environments. Our dataset and benchmark aim to advance indoor scene reconstruction and facilitate the creation of more effective NVS solutions for real-world applications.

## 1. Introduction

### 1.1. Background and Significance

Indoor environments, where people spend a significant portion of their day, are centers of economic activity and value creation. The task of indoor scene reconstruction plays a crucial role in computer vision by enabling the creation of digital replicas of physical spaces. These replicas are essential for numerous applications, including augmented reality (AR), virtual reality (VR), robotic simultaneous localization and mapping (SLAM), and human–environment interaction [1]. The accurate digital replication of physical environments is vital and holds immense significance in the digital era.

For example, in architecture and engineering, the 3D modeling of indoor spaces can provide detailed insights into the internal structure and layout of buildings, leading to cost reductions and enhanced efficiency during construction phases. It can also offer critical information to emergency personnel for response and safety planning, optimize rescue plans, and reduce potential risks [2,3,4]. In the real estate sector, virtual tours enable the remote viewing of properties, providing convenience to both buyers and sellers. In cultural heritage preservation, indoor 3D reconstruction allows for the digital conservation of historical buildings and offers immersive online experiences [5,6].

These diverse applications highlight the demand for accurate novel view synthesis, particularly in indoor settings. Achieving 3D reconstruction through the observation and computation of objects or scenes has been a long-standing task in computer vision. The evolution from traditional methodologies to neural networks has marked significant advancements in this field [7].

Traditional 3D reconstruction algorithms like COLMAP [8,9] have relied heavily on feature extraction and matching techniques, such as SIFT [10] or SURF [11] feature matching, followed by Structure from Motion (SfM) and Multi-View Stereo (MVS) for scene reconstruction. SfM involves extracting keypoints and descriptors from images, matching keypoints across images using descriptors, and estimating camera motion and feature point positions in space when sufficient matched feature points are available. The camera intrinsics and extrinsics, along with the sparse 3D point clouds, are recovered through triangulation and finely adjusted via optimization processes to minimize reprojection errors [8]. MVS, leveraging camera poses obtained through SfM, estimates depth maps for each perspective and fuses them into a consistent dense 3D point cloud [9]. Simultaneous localization and mapping (SLAM) is another technique used for reconstructing 3D environments and obtaining camera poses. SLAM is typically used for real-time applications where a robot or device must navigate and build a map simultaneously. In contrast, SfM or MVS processes are usually performed offline due to their computational intensity. Our dataset utilizes both Lidar-visual-inertial SLAM and SfM software for pose optimization, providing comprehensive ground truth data and ensuring high-quality reconstructions.

However, traditional methods only output a dense point cloud and camera poses, which pose challenges for synthesizing images from new perspectives. Consequently, researchers have explored neural network-based approaches for 3D reconstruction. In 2020, the Neural Radiance Field (NeRF) [12] emerged as a groundbreaking technique, enabling implicit reconstruction results after extensive computation. Despite NeRF’s limitations, such as suboptimal performance, lengthy computation times, and slow rendering speeds, its introduction has significantly impacted the field of 3D reconstruction.

Subsequent works, including Instant-NGP [13] and TensoRF [14], have achieved geometric-level improvements in NeRF’s training and rendering processes, enabling rapid training and high frame rates. Mip-NeRF [15] and its follow-up, Mip-NeRF 360 [16], have enhanced model expressiveness and rendering quality, particularly in unbounded scenes. Other variants, such as DS-NeRF [17], Point-NeRF [18], and Tetra-NeRF [19], have incorporated point clouds or depth supervision, improving performance in sparse perspectives and depth accuracy. Yet, none have achieved photorealistic precision in fine detail rendering for indoor settings.

In 2023, the introduction of 3D Gaussian Splatting [20] marked a significant milestone in the field of novel view synthesis. This model employs a semi-explicit representation, utilizing 3D Gaussian core spheres articulated through spherical harmonic functions to depict color and spatial information. Extensive engineering optimizations in sorting, rendering, and derivation allow for high-precision rendering at super-high frame rates. To further enhance the rendering effects of the 3D Gaussian model, Mip-Splatting [21] adds a filter to remove high-frequency artifacts, ensuring high image quality even after zooming in. SuGaR [22] proposes a method for extracting meshes from 3D Gaussian, which, by binding Gaussians to the mesh surface, enhances rendering capabilities while achieving geometric extraction.

For the algorithms mentioned above, images and their corresponding camera poses are essential inputs for evaluating benchmark datasets. Traditionally, these camera poses are computed using SfM algorithms. Additionally, some NeRF-based algorithms and most 3D Gaussian-based algorithms require point clouds, which are typically generated by MVS algorithms. However, several challenges exist in this data collection pipeline for indoor scenes.

A primary challenge in indoor environments is the presence of repetitive textures and a scarcity of distinctive features. Traditional SfM algorithms heavily depend on identifying and matching features across images. In environments with repetitive patterns, such as wallpaper and floor tiles, feature matching algorithms can mistakenly identify similar textures as identical feature points across different locations. These ambiguities lead to inaccuracies or failures in camera pose estimation. Additionally, smooth surfaces like walls and furniture result in a lack of sufficient feature points, further complicating the extraction of 3D information by SfM algorithms. Both the repetition of patterns and the scarcity of features can cause significant problems. This scarcity of features may also affect NeRF and other NVS algorithms, preventing them from converging properly and achieving accurate reconstructions.

Furthermore, the complex nature of indoor environments, combined with occlusions from furniture and other objects, necessitates capturing images from various angles to obtain a comprehensive view of the scene. This requirement for multi-view capture is particularly challenging to achieve with previous data collection pipelines that rely on handheld front-view mobile phones or DSLR cameras. Additionally, the sparse point clouds generated by SfM often struggle in indoor settings, resulting in low accuracy and high noise levels. These low-quality point clouds can significantly impact the performance of NVS algorithms, leading to inaccurate 3D reconstructions and degraded quality in synthesized novel views.

Recognizing the limitations of SfM alone for indoor environments, we have developed a specialized data collection platform to assemble a large-scale indoor dataset. This platform addresses these limitations and generates the high-quality input data needed for cutting-edge NVS algorithms. Our new dataset introduces the following key features:

Diverse and Challenging Indoor Environments: Our dataset surpasses typical room-scale scenes by including unique and complex indoor spaces. Basements, long corridors, and environments with intricate layouts and occlusions provide a rigorous testbed for NVS algorithms, pushing the boundaries of their capabilities.

High-Resolution, Multi-View Camera System: To capture the rich visual detail and geometric complexity of indoor spaces from as many angles as possible, we employ Insta360 consumer-grade and professional-grade panorama cameras. These cameras provide panoramic coverage, ensuring comprehensive visual information and facilitating robust reconstruction.

LiDAR Sensor: To obtain dense and accurate point clouds that overcome the limitations of SfM-derived point clouds, we integrate an Ouster OS0-128 LiDAR with 128 beams and a 90° vertical FOV into our platform. This setup allows us to generate dense point clouds with a density resolution of up to 5mm.

SLAM Integration: For precise camera pose estimation, especially in challenging, feature-sparse areas, we utilize a robust LiDAR-visual-inertial SLAM algorithm tailored specifically for indoor environments. This integration offers additional pose constraints, resulting in more accurate 3D reconstructions.

### 1.2. Related Work and Comparison

While datasets exist for both indoor scenes and large-scale environments, there is a crucial gap in datasets specifically designed for large-scale, indoor novel view synthesis. As shown in Table 1, indoor datasets are often at a room scale with single camera views, whereas large-scale scene datasets are typically outdoor datasets. Let us examine the landscape below.

#### 1.2.1. Indoor Datasets

Scannet [23]: The ScanNet dataset collected 2.5 million perspectives containing over 1500 different indoor scenes, including apartments, offices, classrooms, bathrooms, and other indoor environments. The dataset provides RGB images, depth images, and corresponding intrinsic and extrinsic camera parameters. Additionally, ScanNet offers rich annotation information, including scene classification, individual labels, and 3D object bounding boxes. The room scale of the ScanNet dataset is small, and the image resolution is relatively low, at only 1296 × 968, with the depth image resolution at 640 × 480.

ScanNet++ [24]: ScanNet++ is a dataset similar to ScanNet, using DSLR and iPhone to capture high-resolution RGB images, and Faro Focus Premium to capture LiDAR data. Although the ScanNet++ dataset captures fewer scenes than ScanNet, it offers higher image resolution and more accurate mesh data. However, its scenes are also small-scale indoor rooms.

ARKitScenes [25]: ARKitScenes was captured by Apple using LiDAR and RGB cameras on an iPad, collecting more than 1900 scenes at 1920 × 1440 resolution. This is the largest indoor dataset and the accuracy is satisfactory. However, there is still the issue of small scale for individual scenes.

MuSHRoom [26]: The ’MuSHRoom’ dataset addresses immersive real-time modeling using consumer-grade hardware for non-human perception and AR/VR. It offers room-scale 3D reconstruction and novel view synthesis data from multiple sensors (Azure Kinect, iPhone, and laser scanner). The dataset tackles challenges such as occlusion, motion blur, and illumination diversity, aiming to enhance 3D reconstruction and rendering.

#### 1.2.2. Large-Scale Scene Datasets

Tank and Temples [27]: The Tank and Temples dataset is one of the earliest large-scale scene datasets, captured using high-resolution cameras. It includes camera poses reconstructed using SfM/MVS algorithms (COLMAP), with some scenes featuring true point clouds and their corresponding relationships. However, the dataset’s indoor scenes are relatively small in scale, and the larger scenes with point clouds are all outdoors.

GigaMVS [28]: GigaMVS features scenes from the Summer Palace captured with an ultra-high-resolution camera, covering an area of over 40,000 square meters. However, this dataset is limited to outdoor scenes.

KITTI 360 [29]: KITTI-360 is a large-scale outdoor dataset designed for autonomous driving research. It includes omnidirectional RGB images and uses LiDAR and radar to capture surrounding point clouds. Additionally, it provides rich labels for various objects. Despite these features, the images in the KITTI 360 dataset are low in resolution, and the visible range is limited.

GauU-Scene [30]: The GauU-Scene V2 dataset combines highly accurate LiDAR point clouds with comprehensive RGB images. It surpasses existing datasets in both area and point count, covering over 6.5 square kilometers. The authors propose a novel method to align LiDAR and image data, addressing discrepancies in coordinate systems. They evaluated various reconstruction methods, highlighting limitations in current image-based metrics and emphasizing the importance of reliable ground truth for geometry reconstruction tasks.

#### 1.2.3. Datasets for Novel View Synthesis

As mentioned earlier, many novel view synthesis (NVS) schemes are available, often tested in idealized settings, coming up with their own datasets. These include cameras arranged in hemispherical or cylindrical configurations, as seen with NeRF synthetic data or Mip-NeRF 360 data [16], or forward-facing views similar to LLFF [31]. Some autonomous driving research also uses datasets like KITTI-360 [29] for NVS, where images follow the direction of the road. Typically, these algorithms use small road segments for experiments. From a practical perspective, it is desirable for NVS to be applicable to large-scale indoor and outdoor scenes. Implicit representations are challenging to split and merge compared to explicit representations. However, block processing based on semi-explicit Gaussian representations is relatively simple. Despite the achievements in large-scale scene NVS, there is a lack of suitable public datasets for testing. Consequently, representative works such as Block-NeRF [32], Mega-NeRF [33], and VastGaussian [34] have collected their own datasets. Although their work is commendable, the lack of open-sourced datasets limits the development of other related work.

Currently, the field of SLAM (simultaneous localization and mapping) has witnessed numerous mature benchmark efforts [35,36,37]. Meanwhile, existing indoor datasets and benchmarks for novel view synthesis (NVS) predominantly suffer from limitations in scale and the absence of comprehensive ground truth data. Notably, they exhibit the following shortcomings:

**Limited Scene Scale**: Most available datasets feature small-scale scenes that do not adequately capture the complexity and diversity of real-world environments.

**Scarcity of High-Quality Indoor Data**: High-quality indoor datasets are rare, and those available do not offer high-resolution cameras or point clouds. Even when such data exist, it is typically derived from SLAM datasets, which are restricted to unidirectional paths and lack panoramic coverage.

**Sparse Viewpoints**: The provided viewpoints are often too few, which limits the ability to perform robust NVS. This is particularly problematic in indoor settings, where multiple angles are crucial for understanding the spatial layout.

**Absence of Ground Truth Point Clouds**: There is a significant lack of datasets providing ground truth point clouds, essential for accurate depth estimation and 3D reconstruction.

These limitations have significantly hindered progress in large-scale NVS and the application of indoor scene reconstruction. Many studies have been constrained to using small-sized datasets like ScanNet, which do not provide the necessary scope for advancing the field. In summary, existing datasets lack a comprehensive benchmark for large indoor scenes, which includes full-view clear images, accurate corresponding camera poses, and Lidar data. Therefore, we propose the IVGM dataset to address this gap.

This work introduces a comprehensive dataset and benchmark designed to address the aforementioned limitations, with the following contributions:

- We present the first large-scale indoor dataset featuring multiple diverse and challenging scenes, such as basements and long corridors, to better simulate real-world conditions. This makes it a robust tool for developing and testing novel view synthesis (NVS) algorithms, addressing the limitations of limited scene scale and the scarcity of high-quality indoor data.
- Our dataset provides panoramic views captured by a multi-camera system, offering extensive coverage and facilitating complete scene understanding. This setup incorporates both consumer-grade and professional-grade panorama cameras, ensuring extensive coverage and high-quality visual data, overcoming the issue of sparse viewpoints.
- We provide high-quality ground truth point clouds accompanied by detailed textured meshes, which enable precise depth perception and realistic NVS. The inclusion of both point clouds and meshes supports a wide range of NVS methods and applications, which compensates for the absence of ground truth point clouds.
- We introduce a thorough benchmark and conduct extensive experiments to evaluate the performance of NVS algorithms. This benchmark rigorously tests the efficacy of these algorithms in large-scale and complex indoor settings, providing valuable insights and driving advancements in the field.

## 2. IVGM Dataset

### 2.1. Collection Platform

**Figure 1:**
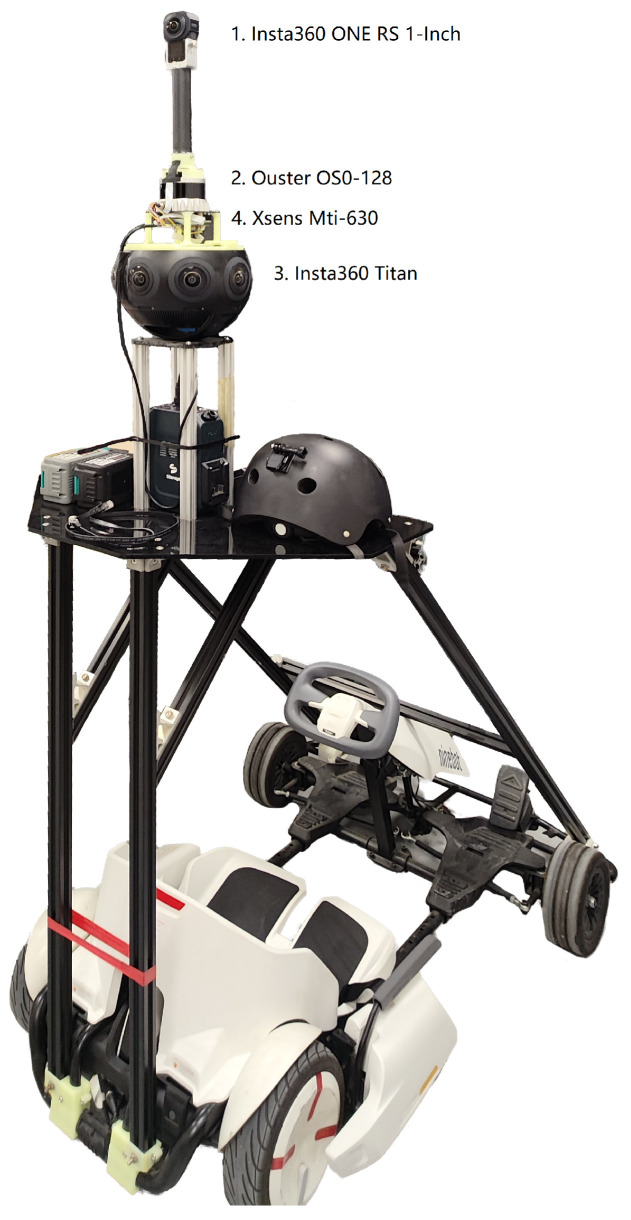
Go-Kart data collection platform equipped with Ouster OS0-128 LiDAR and Insta360 cameras.

To assemble our dataset, we employ a comprehensive set of sensors, detailed in Table 2 and Figure 1. These include the Ouster OS0-128 LiDAR, known for its wide field of view and high point density; the Insta360 ONE RS 1-Inch 360 Edition camera, which provides high-resolution 360-degree imagery; and the Insta360 Titan camera, capable of capturing professional-grade 11K panoramic videos. We have customized an electric Go-Kart to house these sensors, enabling efficient data collection in both indoor and outdoor environments.

The Ouster OS0-128 LiDAR, combined with the Insta360 ONE RS 1-Inch 360 Edition camera, is adapted from the Stereye Polar 3D Scanner. This setup includes an Xsens Mti-630 IMU, which supports precise LiDAR-Inertial-Visual odometry, enabling real-time environmental scanning and previews on mobile devices. The Stereye Polar post-processing software streamlines the workflow by extracting essential data, including LiDAR and ONE RS camera poses, colored point clouds, textured meshes, and raw RGB and LiDAR data.

The Ouster OS0-128 REV6 is an ultra-wide field-of-view LiDAR sensor, featuring a 90° vertical FOV and a 35-meter range at a 10% reflectivity. With 128 channels, it outputs up to 5.2 million points per second. Powered by the L2X digital LiDAR chip, this sensor can output up to 5.2 million points per second, capturing both the strongest and second-strongest light returns for each point [38].

The Insta360 ONE RS 1-Inch 360 Edition is a consumer-grade 6K 360 camera, featuring dual 1-inch Sony IMX283 rolling shutter sensors. These large sensors capture 3072 × 3072 fisheye images, enhancing light capture and detail for higher-quality images and videos with reduced noise [39].

The Insta360 Titan, on the other hand, is a professional-grade 360 camera that records 11K panoramic videos. It features eight Sony IMX269 Micro-4/3″ sensors, known for their high image quality. These sensors, typically used in DSLR cameras, contribute to the Titan’s exceptional image and video output [40].

In our setup, the cameras offer limited manual control, primarily relying on automatic settings with fixed exposure times and ISO values. Each camera has specific thresholds for ISO and exposure time to maintain optimal image quality. For instance, the ONE RS produces excessive noise above ISO 800, while the Titan becomes noisy above ISO 1600. Additionally, exposure times shorter than 1/100 s typically avoid motion blur. We conducted tests under various lighting conditions to adjust these settings accordingly. In well-lit environments, both motion blur and significant noise are minimized, resulting in good image quality across different sensor configurations. However, in low-light conditions, adjusting the settings can introduce either motion blur or noise. To ensure the highest-quality data, we carefully selected the best settings for each scenario. For synchronization and storage efficiency, we recorded in video mode, with the Titan operating at 5 Hz and the ONE RS at 24 Hz, then extracted frames from the videos and assigned timestamps for further processing.

For large-scale scenes, our primary goal is to avoid relying solely on traditional Structure-from-Motion (SfM) to obtain camera poses due to its limitations, as we aim to achieve the highest precision in pose estimation. To synchronize the video of Insta360 Titan camera and the ONE RS camera, manual alignment is performed using distinct events, such as clapping, captured at the beginning and end of the video. The calibration of intrinsic and extrinsic parameters for both cameras is carried out using Kalibr [41]. This process provides the intrinsic parameters for each fisheye lens and the necessary extrinsic parameters both between the lenses within one camera and between the cameras. With the initial poses from the ONE RS camera and the extrinsic, we estimate the initial poses of the Titan camera. For large-scale scenes, to further refine the accuracy of the camera poses, Agisoft SFM software is employed. The initial camera poses, derived from LiDAR-Inertial-Visual odometry, serve as constraints within the Agisoft SFM framework to enhance the precision of the pose estimates. Additionally, advanced methods like CP+ [42] may be applied for further refinement and improved accuracy in camera pose estimation.

### 2.2. Dataset Content

The IVGM dataset encompasses a diverse array of environments, meticulously captured by our custom-designed data acquisition vehicle across three distinct scenes. Shown in Table 3, this collection includes two segments from school office floors and one scene from underground garages.

**Figure 2:**
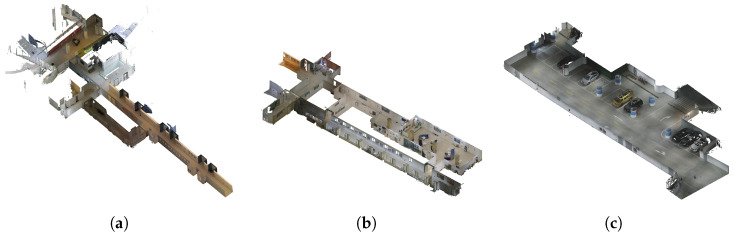
Three dataset sequence ground truth mesh view. (**a**) Office Area 1. (**b**) Office Area 2. (**c**) Underground Garage.

**Office Area 1:** As depicted in Figure 2a, this area features long indoor corridors, a public zone with floor-to-ceiling windows overlooking an elevator, and a transparent glass platform offering a view of the atrium.

**Office Area 2:** Illustrated in Figure 2b, this area includes a series of indoor corridors, an outdoor corridor, a public rest area, administrative offices, and a designated tea break area for relaxation and informal interactions.

**Underground Garage:** The Underground Garage shown in Figure 2c is located under ShanghaiTech University. To achieve a higher density of visual perspectives, our data capture process involved multiple passes along the main thoroughfare. Additionally, to ensure optimal and uniformly distributed lighting, all lighting fixtures within the garage were activated during the capture process.

Due to the significant reliance on natural lighting in Office Areas 1 and 2, our tests revealed that, regardless of camera settings, there were substantial issues with overexposure and underexposure during both daytime and nighttime. Consequently, we opted to conduct data collection during the early morning hours, when the lighting is softer. Additionally, this time frame experiences relatively less human and vehicle traffic, allowing for more effective observation and recording.

### 2.3. Challenges of IVGM Dataset

The IVGM dataset is captured in real-world conditions, presenting many complex situations not found in ideal datasets. These include significant exposure changes, reflections, transparent objects, motion blur, and a small number of dynamic objects.

Large Scene Area and High Perspective Density: Ideal test data for neural network visual reconstruction and novel view synthesis based on implicit representation typically involve small-scale, “object-centric” scenes with a central point of interest and uniformly distributed perspectives, using about 200 to 300 images. Each training image usually includes this central point, resulting in significant overlap between images. However, for large scenes, a central point does not exist, requiring the network to have robust expression and fitting capabilities. Specifically, in large scenes, the pose estimation using traditional methods like SfM alone requires an enormous amount of time and computational resources. Additionally, SfM often encounters problems such as scale ambiguity and inaccuracies in pose determination. Moreover, large scenes contain a significant number of images, and storing point clouds requires substantial space. This demands advanced engineering capabilities to manage and process the extensive data efficiently.

Large Changes in Light: The IVGM dataset contains multiple real large-scale indoor scenes with complex lighting conditions. In Office Areas 1 and 2, sunlight shines through large glass windows. Although the data were collected during optimal times to minimize overexposure, significant differences between natural and artificial light are still evident in the images. In the Underground Parking Garage, all lights were turned on to ensure uniform brightness, though some areas remained dim. Learning-based NVS algorithms are highly sensitive to light variations, which are inevitable in large scenes. Therefore, managing light changes and addressing noise in low-light conditions, potentially through the use of denoising algorithms, presents a significant challenge.

Motion Blur: The collection platform we designed can be driven stably on smooth roads. In addition, we have limited the speed to 1 m/s. Despite adjusting the exposure time optimally based on the environment, the need to use automatic settings for shutter speed and white balance due to changing lighting conditions results in some unavoidable motion blur. Furthermore, vibrations and significant rotations during recording contribute to this blur. While this poses a challenge for the learning capability of NVS algorithms, the use of deblurring algorithms could potentially improve image quality in these situations.

Reflections and Transparent Objects: Indoor scenes often feature transparent and reflective surfaces, such as glass and metal. In Office Areas 1 and 2, windows, glass walls, and glass doors create transparency, while frames, elevators, and handrails add reflective properties. These elements pose challenges for both LiDAR and the camera in relation to accurate data capture and novel view synthesis. Our previous work [43,44] addresses these challenges by developing methods for detecting reflections in 3D LiDAR scans. Incorporating these techniques into our current dataset may help mitigate the issues posed by reflective and transparent surfaces, leading to more accurate reconstructions and improved novel view synthesis.

## 3. Experiments

### 3.1. Evaluation of Algorithms

To evaluate the applicability, versatility, and performance of our dataset on novel view synthesis algorithms, we tested several popular methods developed in recent years. We categorized the algorithms into two groups: those requiring geometric information and those using only images as input.

Algorithms requiring only images as input: These algorithms are usually modifications based on NeRFs.

NeRFStudio [45] offers researchers a robust algorithm framework, integrating many existing algorithms. Since its open-source release, many projects have been developed using its framework. The inputs for most algorithms within the NeRFStudio framework are standardized. Additionally, NeRFStudio introduces an extension algorithm: Nerfacto. Thus, we selected the NeRFStudio framework and its implemented algorithms for our tests.

Instant-NGP [13] introduces multi-resolution hash grid into a NeRF and implements a lot of engineering optimization, significantly reducing the training time of the NeRF to mere seconds.

Nerfacto [45] was proposed by the author of NeRFStudio. It integrates numerous previous works based on NeRF, altering the ray generation and sampling methods as well as the scene contraction method. Nerfacto offers three models of different scales: nerfacto, nerfacto-big, and nerfacto-huge. We conducted experiments with nerfacto and nerfacto-huge models.

Algorithms requiring geometric input: These algorithms typically use depth images or point clouds as geometric priors. We selected two representative algorithms.

READ [46] takes images and point clouds as inputs and proposes a rendering network. Using Monte Carlo sampling and multi-scale feature fusion, it performs novel view synthesis based on autonomous driving tasks.

3D Gaussian Splatting [20] uses images and point clouds as inputs, employing them as the basis for 3D Gaussian spheres. It utilizes spherical harmonics to represent anisotropic colors and applies backpropagation to optimize their position, color, and shape.

### 3.2. Data Preprocessing

One of the primary challenges in data processing is the adaptation of fisheye images. Common NVS algorithms and datasets typically employ consumer-grade smartphones or cameras to record from a single viewpoint. To simulate this scenario, we selected only the front fisheye camera for the consumer-grade Insta360 One RS camera. Most current NVS algorithms primarily support the pinhole camera model. The two types of camera image data provided in our dataset are both fisheye and distorted. To make our dataset more relevant and easily applicable to the majority of existing NVS methodologies, we have developed a preprocessing pipeline that transforms the distorted fisheye images into three types of data:

Insta ONE RS Camera Single View: We simply undistorted the fisheye images using the OpenCV fisheye model to pinhole image. The images were undistorted to a width and height of 1000 pixels each, with a field of view of 90 degrees. This approach crops out the surrounding perspective distortion while retaining more scene content.

Insta ONE RS Camera Five View: The Insta ONE RS camera has fisheye lenses that capture a wider field of view than pinhole lenses, providing more angular information. Directly undistorting the fisheye image to single view can lead to substantial loss of captured details. To address this, we have adopted a similar approach as outlined in the paper [47]. We projected the fisheye image onto a virtual hemisphere, then onto a virtual pinhole camera. We set up five virtual cameras facing up, down, left, right, and front, respectively. The width, height, and field of view of each camera match the previous fisheye "undistort" settings, so the image of the forward camera is identical to the single-view undistorted result.

Insta Titan Camera: There is only a small amount of wide-angle distortion in the images of the Insta Titan camera, so we chose to undistort the images using the fisheye model. The processed images have a width of 999 pixels, a height of 1776 pixels, and a horizontal field of view of 90 degrees.

**Figure 3:**
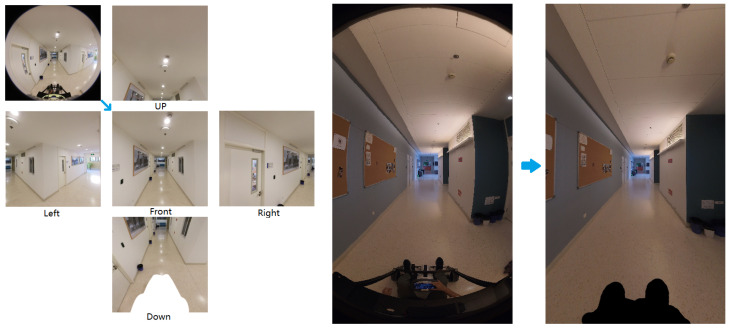
Comparison of original and processed images for fisheye undistortion using Insta360 One RS (**left**) and Insta360 Titan (**right**) cameras.

In some images, the devices used to capture the data are visible, so we manually added masks to these images. Figure 3 shows the result of the original and processed images. We also preprocessed the collected point clouds, downsampling them to a spacing of 3 cm to reduce GPU memory consumption.

### 3.3. Implementation Details

All training hyper-parameters followed the original paper’s settings in our experiments. Each scene was trained on a single Nvidia 3090 GPU for approximately 3–22 h, depending on the method’s time complexity, with 4× Nvidia 3090 GPUs used in parallel. Due to the large number of images in the large scenes, we have 600 GB RAM per device.

For the Nerfstudio methods, we trained without modifying the parameters. For READ, based on the official website guidance, we changed the *–crop_size* to 448 × 448 according to our memory capacity. For 3D Gaussian Splatting, following the author’s instructions for a large dataset, we set *–position_lr_init* to 0.000016 and *–scaling_lr* to 0.001, and increased the number of iterations to 20–30 times the number of images. All methods enabled the image mask.

### 3.4. Evaluation Metrics

To evaluate the performance of each method, we used three common metrics: Peak Signal-to-Noise Ratio (PSNR), Structural Similarity (SSIM) [48], and Learned Perceptual Image Patch Similarity (LPIPS) [49] for novel view synthesis. The interpretation of these metrics is straightforward: higher PSNR and SSIM scores suggest superior image quality, whereas a lower LPIPS score denotes a more accurate perceptual match to the original scene.

PSNR: Peak Signal-to-Noise Ratio measures the quality of reconstructed images. It is calculated by averaging the squared pixel differences between the original image and the reconstructed image, followed by taking the logarithm.

SSIM: Structural Similarity assesses the structural similarity between two images. It considers luminance, contrast, and structural information. Higher SSIM values indicate better perceptual similarity.

LPIPS: Learned Perceptual Image Patch Similarity quantifies the perceptual difference between images. It leverages deep neural networks to capture perceptual features. Lower LPIPS scores indicate better perceptual similarity.

These metrics provide valuable insights into the quality and perceptual fidelity of synthesized views, aiding in the evaluation and comparison of different methods.

## 4. Benchmark

### 4.1. Benchmark for Different Algorithm on Different Sensors

**Figure 4:**
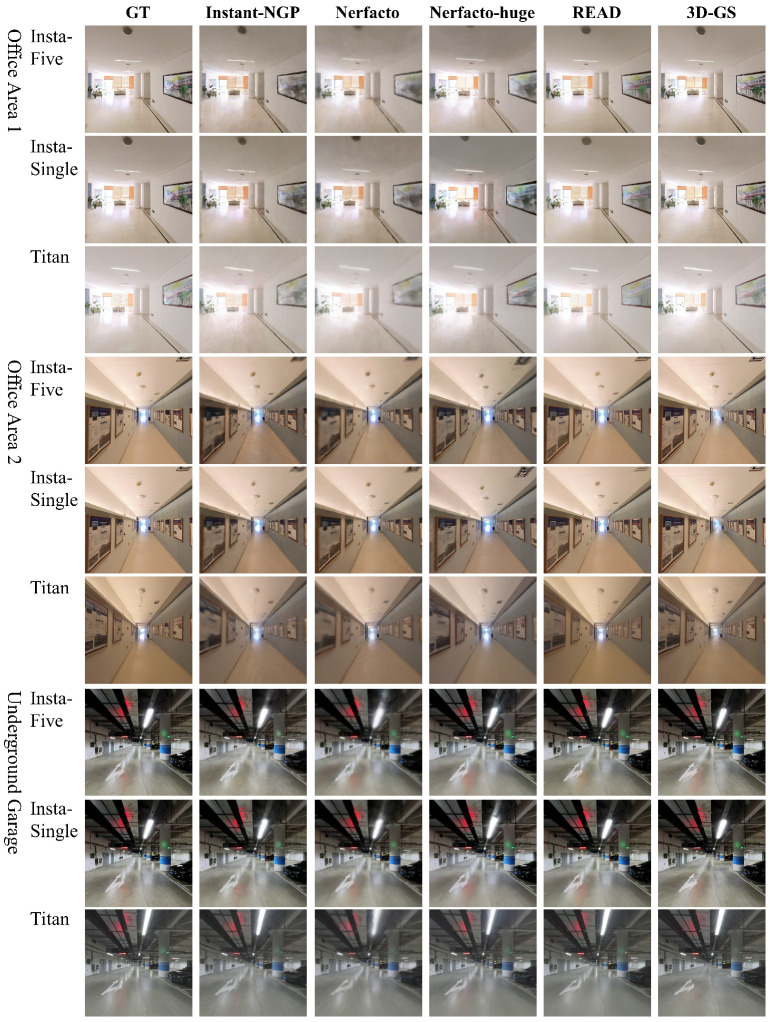
Qualitative comparison of different methods on the IVGM dataset. The Titan result images are cropped to an Insta image size.

Table 4 and Figure 4 show the results of various novel view synthesis (NVS) algorithms when applied to images captured by different sensors.

The Gaussian Splatting method consistently outperforms others across all scenes, as evidenced by the highest PSNR and SSIM values and the lowest LPIPS scores. This highlights the effectiveness of the semi-explicit representation used by Gaussian Splatting in rendering high-quality images in complex indoor environments. Instant-NGP achieves commendable results, particularly with the Insta360 Titan camera, showing significant improvements in PSNR and SSIM when higher-resolution images are used. For both Nerfacto and Nerfacto-huge, performance improves with higher-resolution images from the Titan camera, although Gaussian Splatting still leads overall. Nerfacto-huge, despite its larger model size, performs slightly worse than the smaller Nerfacto model, potentially due to the difficulty in converging larger models in complex indoor scenarios. READ exhibits variable results, performing well in certain scenarios but not consistently across all camera settings.

The results also indicate that the choice of camera system can significantly affect the performance of NVS algorithms. For instance, higher-resolution and multi-view sensors, such as the Insta360 Titan camera, generally enhance the performance of NVS algorithms, highlighting the importance of image quality in these applications.

### 4.2. Cross-Camera Evaluation

**Insta One Camera Single View vs. Five View**

Table 5 presents a cross-camera evaluation of NVS algorithms using a single image versus five images from the Insta360 ONE RS 1-Inch 360 Edition camera. Novel view synthesis (NVS) algorithms strive to fit the training images as closely as possible. Consequently, when only the frontal view is provided, images from other directions (left, right, up, and down) lack a default perspective and are typically excluded from evaluation. However, the five-view setup includes the frontal perspective, which is a comparison demonstrating the impact of multiple viewpoints on algorithm performance.

The evaluation shows that models trained using images from the frontal camera render noticeably lower-quality results on the five-camera view compared to rendering on their respective training sets. In contrast, models trained with images from all five cameras better fit the images from all perspectives. The render results of the front view are not only similar to all views but also significantly better than those from single-camera training. This indicates that synthesizing views from unobserved angles, or free-view synthesis, can severely impact rendering quality. Our five-view preprocessing method is effective and improves overall performance.

**Insta Camera vs. Titan Camera**

**Figure 5:**
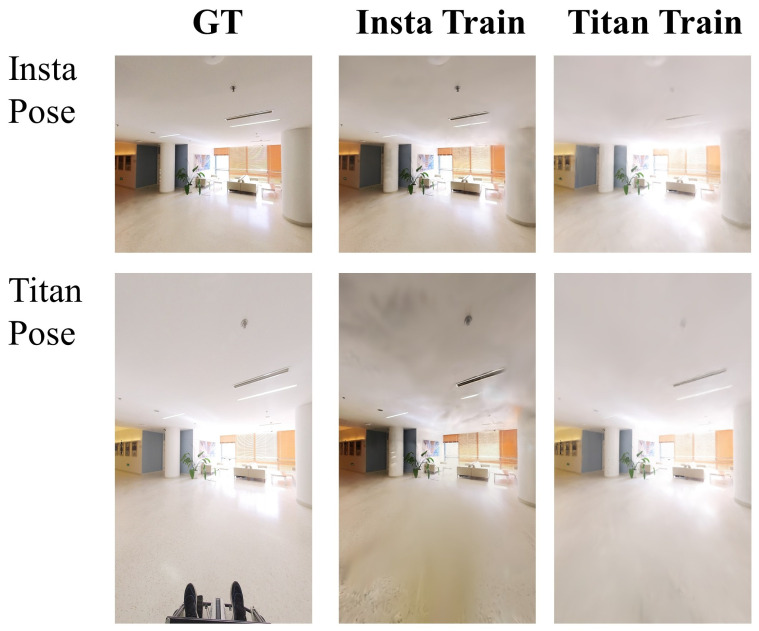
Qualitative comparison of Gaussian Splatting render on Insta and Titan camera poses.

The Insta360 ONE RS (Insta) and Insta360 Titan (Titan) cameras are both oriented towards the front, each with a horizontal field of view (FOV) of 90 degrees. However, the Insta camera is positioned approximately 60 cm above the Titan camera. To compare the frontal view rendering effects of the two cameras, we rendered images from each camera in each other’s pose using the Gaussian Splatting method. Figure 5 shows that the Titan camera, which captures a larger number of images and has a wider field of view, significantly reduces noise in the reconstructed results. Additionally, the varying color tones among the different camera groups provide a rich dataset for future research in multi-camera style transfer.

### 4.3. Point Cloud Effect

**Figure 6:**
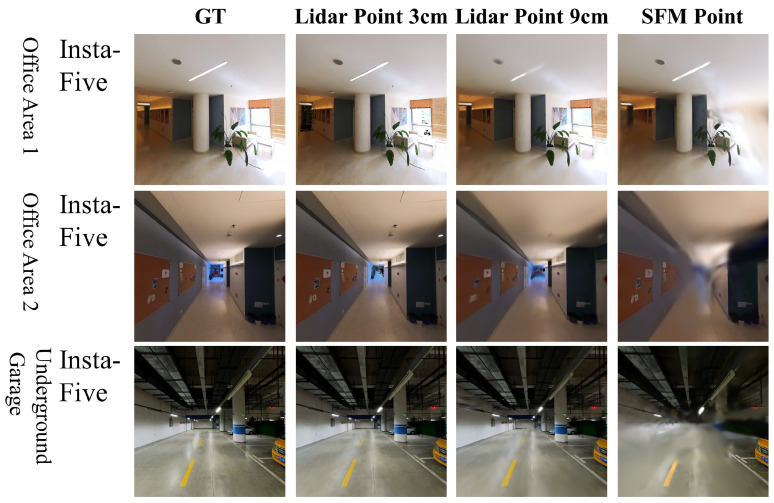
Qualitative comparison of Gaussian Splatting with different input point clouds.

We applied the Gaussian Splatting algorithm to sparse Structure-from-Motion (SfM) point cloud and LiDAR point cloud data, which are downsampled to spacings of 3 cm and 9 cm, respectively. Insta-five images are used for all settings and training iterations are set to 200,000. Figure 6 and Table 6 illustrate the impact of integrating LiDAR point cloud data with images using the Gaussian Splatting algorithm. The inclusion of LiDAR point cloud data significantly enhances the performance of the novel view synthesis (NVS) algorithm, resulting in higher PSNR and SSIM values and lower LPIPS, compared to using only image data or sparse point clouds from SfM. In addition, denser point clouds also boost performance. These results indicate that high-quality point cloud data, such as those from LiDAR, are crucial for improving the accuracy and quality of novel view synthesis, especially in complex indoor environments.

### 4.4. Training Time Consumption

Due to the varying CPUs and storage devices used in our training equipment, there may be slight differences in training times. Therefore, we have provided a summary of the approximate training times in Table 7.

Instant-NGP and Nerfacto still have a significant advantage in training speed. Although Nerfacto-huge and READ require longer training times, their quality does not match that of Gaussian Splatting, making them relatively less competitive solutions.

### 4.5. Storage Consumption

Table 8 shows the storage space required by different methods. The methods based on NeRFStudio are stored in .ckpt file format, while READ consists of two .pth files: PointTexture Checkpoint and UNet Checkpoint. The Gaussian Splatting models are saved in .ply format. It is important to note that the version of NeRFStudio we used is 0.3.4, and the size of .ckpt files may vary in updated versions. For comparison, we also listed the file sizes of the original point cloud we collected (with a minimum interval of 5 mm) and the downsampled point cloud with a 3 cm interval.

It is shown that for methods based on NeRFStudio, the storage differences between different datasets are relatively small, with variations in size primarily arising from the differences in methodologies. The Nerfacto-huge method, which has the largest number of parameters set in the network, also requires the longest optimization time and the most storage space. The parameter count for the READ UNet checkpoint remains constant, resulting in the same .pth file size across different datasets. Meanwhile, the number of parameters for PointTexture is positively correlated with the size of the point cloud.

For Gaussian Splatting, there is a significant difference in storage requirements between groups, which generally correlates with the scale of the scene. Additionally, the more viewpoints provided, the more Gaussian kernels are generated in the space to fit complex scenes, leading to greater storage demands. Considering both storage consumption and image quality, we can conclude that Gaussian Splatting remains the optimal solution among the various approaches.

## 5. Discussion

The IVGM dataset represents a significant advancement in the field of novel view synthesis (NVS) and indoor scene reconstruction. By addressing the limitations of existing datasets, it provides a robust and comprehensive benchmark for large-scale indoor environments. A notable contribution of this dataset is its inclusion of diverse and challenging scenes, such as basements and long corridors, which offer a more accurate reflection of real-world conditions than the often idealized settings of previous datasets.

The use of a multi-camera system to capture panoramic views significantly enhances the dataset’s utility by ensuring extensive coverage and facilitating a comprehensive understanding of the scene, which is vital for NVS algorithms. The dataset includes images from both consumer-grade and professional-grade cameras, supporting a wide array of research applications and ensuring versatility.

Reconstruction results obtained with the industrial-grade Titan camera outperform those achieved with the consumer-grade Insta ONE RS camera, mainly due to the Titan’s broader field of view, lower noise levels, and higher image quality. Despite this, the Titan camera’s higher tolerance for exposure bias makes it prone to overexposure—a drawback for image-based reconstruction. Future research may focus on harmonizing the exposure and color profiles of both cameras to enable the simultaneous input of images from both, facilitating more comprehensive reconstructions. The experiment also demonstrates that undistorting large FOV fisheye images into five separate images retains more information and results in better reconstruction quality. Although this five-view undistortion may also lead to some information loss, researchers should explore direct training on fisheye models to achieve better results.

The high-quality ground truth point clouds, detailed meshes, and textures provided by the IVGM dataset are significant strengths, enabling precise depth perception and realistic NVS, crucial for applications in robotics, virtual reality, and augmented reality. However, methods like Gaussian Splatting have not fully utilized the high-quality LiDAR point clouds, using them only for initialization. The better utilization of these high-quality point clouds could enhance rendering quality significantly.

Training models on datasets that encompass extremely large scenes presents a significant challenge, particularly in the domain of Neural Visual Synthesis (NVS), especially when constrained by limited hardware resources. To address this issue, some researchers [32,34,47,50] have opted to segment the data into smaller chunks, training each segment independently before merging the results. Others have taken a more engineering-focused approach, developing distributed training systems to manage the workload effectively. These methodologies often rely on separately collected datasets, a necessity driven by the current absence of a high-quality, universal dataset and benchmark for the field. We aim for our work to contribute to addressing this gap, providing a more cohesive dataset that can serve as a resource for researchers. By doing so, we hope to facilitate advancements in future research efforts within this area, ultimately promoting the development of more effective and efficient training techniques for NVS.

As mentioned earlier, the panoramic camera we used exhibited issues such as overexposure, underexposure, and motion blur in relatively poor lighting conditions. Additionally, it does not support capturing images in RAW format, which limited our ability to take multiple shots of the same scene under varying lighting conditions. In our future work, we plan to upgrade the camera and attempt to capture fixed video recordings to facilitate multiple acquisitions of the same scene. We will also utilize our current or improved methods to capture more extensive and larger scenes, continuously updating our dataset in a manner similar to ScanNet [23].

## 6. Conclusions

We introduce a novel data acquisition device capable of simultaneously capturing high-precision LiDAR point clouds and two sets of high-resolution images. This device provides accurate camera parameters for the captured images. Using this equipment, we have collected a diverse array of large-scale indoor scenes within a campus environment, including office spaces and underground garages, resulting in the first large-scale indoor campus environment dataset.

The IVGM dataset sets a new standard for indoor visual–geometric datasets by addressing significant gaps in scale, quality, and comprehensiveness present in existing datasets. Its inclusion of large-scale, diverse, and challenging indoor scenes, coupled with high-resolution panoramic imagery and detailed ground truth data, makes it an invaluable resource for advancing the fields of NVS and indoor scene reconstruction.

We have also established a benchmark for indoor reconstruction and conducted experiments with several state-of-the-art novel view synthesis methods. Our findings indicate that existing methods can successfully perform reconstruction and new view synthesis on our dataset, with high-precision point clouds enhancing the quality of results for methods relying on point clouds as input. We anticipate that our dataset will serve as a valuable resource for future research in large-scale indoor reconstruction and generation.

In conclusion, the IVGM dataset addresses the critical need for a comprehensive indoor visual–geometric dataset and benchmark, paving the way for future research and development in novel view synthesis and beyond.

## Figures and Tables

**Table 1:** Dataset comparison. I/O denotes indoor or outdoor datasets.

I/O	Dataset	Avg Size	Device	Resolution	Pose	GT
I	Scannet	22.6 m²	iPad Air2 Occipital Structure Sensor	1296 × 968	Hardware	Mesh
I	Scannet++	32.6 m²	Sony A7IV iPhone13 pro Faro laser scanner	1920 × 1440	COLMAP	Mesh Point
I	ARKitScenes	<200 m²	2020 iPad Pro Faro Focus S70	1920 × 1440	Hardware	Point
I	MuSHRoom	61.4 m²	Azure Kinect iPhone 12 Pro Max Faro Focus X130	1280 × 720 994 × 738	COLMAP	Mesh
O	Tanks and Temples	462 m²	DJI X5R Sony a7S II Faro Focus X330	3840 × 2160	COLMAP	Point
O	GIGAMVS	8667 m²	Nikon Camera Sony A7R4 Rigel VZ400	gigapixel	COLMAP	Point
O	KITTI360	73.7 km	2x fisheye camera Velodyne HDL-64E SICK LMS 200	1440 × 1440	OXTS and RTK	Point
O	GauU-Scene	1.1 km²	DJI Zenmuse L1	5472 × 3648	COLMAP	Point
I	Ours	3145 m²	Insta360 Dual 1-Inch Insta360 Titan Ouster OS0-128	3072 × 3072 2972 × 5280	SLAM and SFM	Point Mesh

**Table 2:** Sensors specifications.

#	Sensor	Modalities	FOV	Resolution	FPS
1	Insta360 ONE RS 1-Inch	2× 1“ Fisheye Camera	180° × 180°	3072 × 3072	24
2	Ouster OS0-128	Spinning LiDAR	90° × 360°	128 × 1024	10
3	Insta360 Titan	8x M43 Fisheye Camera	180° × 70°	2972 × 5280	5
4	Xsens Mti-630	IMU	-	-	400

**Table 3:** Dataset sequence statistics.

Sequence Name	Area Size (m²)	Point Number	Insta Images	Titan Images
Office Area 1	2989.63	76,488,066	1610	12,872
Office Area 2	2651.00	86,233,513	2669	21,608
Underground Garage	3797.11	153,185,271	1816	14,528

**Table 4:** Quantitative comparison of different novel view synthesis methods in the IVGM dataset. The results for each scene are independently ranked and the top two values are highlighted. Red indicates the highest and Orange indicates the second highest.

Method		Office Area 1			Office Area 2			Underground Garage		
		**Single**	**Five**	**Titan**	**Single**	**Five**	**Titan**	**Single**	**Five**	**Titan**
Instant- NGP	PSNR↑	22.35	22.68	24.66	25.85	25.97	26.69	24.90	23.67	28.69
	SSIM↑	0.840	0.821	0.885	0.901	0.788	0.910	0.804	0.753	0.907
	LPIPS↓	0.334	0.352	0.299	0.308	0.416	0.318	0.378	0.429	0.384
Nerfacto	PSNR↑	19.48	20.22	24.07	23.67	23.01	25.76	24.35	23.66	28.10
	SSIM↑	0.782	0.784	0.874	0.873	0.850	0.901	0.779	0.747	0.900
	LPIPS↓	0.368	0.375	0.308	0.330	0.359	0.327	0.412	0.453	0.403
Nerfacto- huge	PSNR↑	18.12	18.72	21.65	21.06	20.79	23.89	23.37	23.13	25.73
	SSIM↑	0.758	0.766	0.859	0.843	0.829	0.891	0.797	0.775	0.893
	LPIPS↓	0.379	0.377	0.313	0.345	0.369	0.336	0.340	0.389	0.362
READ	PSNR↑	27.12	19.07	22.38	27.75	19.99	20.48	27.63	22.68	22.89
	SSIM↑	0.873	0.783	0.866	0.884	0.789	0.835	0.845	0.732	0.861
	LPIPS↓	**0.212**	0.383	0.285	0.239	0.382	0.338	0.264	0.362	0.357
Gaussian Splatting	PSNR↑	28.42	28.09	**28.84**	31.32	**32.27**	31.27	28.04	29.87	**30.00**
	SSIM↑	0.899	0.870	**0.909**	0.912	**0.938**	0.930	0.862	0.892	**0.921**
	LPIPS↓	0.242	0.282	0.253	0.280	**0.235**	0.277	0.203	**0.151**	0.223

**Table 5:** Quantitative results of different input image datasets on the IVGM dataset. Images of Insta-single results are rendered using Insta-five camera poses and vice versa.

Method	Camera Path	Office Area 1		Office Area 2		Underground Garage	
	**Training Set**	**Single**	**Five**	**Single**	**Five**	**Single**	**Five**
	**Rendering Set**	**Five**	**Single**	**Five**	**Single**	**Five**	**Single**
Instant- NGP	PSNR↑	21.41	22.87	23.59	26.39	22.75	23.99
	SSIM↑	0.813	0.837	0.869	0.898	0.751	0.778
	LPIPS↓	0.357	0.346	0.346	0.317	0.425	0.402
Nerfacto	PSNR↑	19.26	20.19	22.44	23.43	22.77	23.96
	SSIM↑	0.770	0.789	0.845	0.866	0.739	0.765
	LPIPS↓	0.379	0.379	0.358	0.349	0.445	0.432
Nerfacto- huge	PSNR↑	18.05	18.81	20.46	21.25	21.96	23.30
	SSIM↑	0.747	0.769	0.819	0.845	0.752	0.791
	LPIPS↓	0.390	0.375	0.371	0.355	0.399	0.353
READ	PSNR↑	24.00	18.99	23.93	20.34	24.19	23.41
	SSIM↑	0.826	0.806	0.844	0.806	0.780	0.758
	LPIPS↓	0.333	0.333	0.326	0.348	0.351	0.321
Gaussian Splatting	PSNR↑	23.34	**27.50**	26.17	**31.28**	23.03	**27.19**
	SSIM↑	0.826	**0.869**	0.874	**0.930**	0.753	**0.859**
	LPIPS↓	0.316	**0.260**	0.318	**0.244**	0.235	**0.178**

**Table 6:** Quantitative results of Gaussian Splatting with different input point clouds.

	LiDAR-3 cm			LiDAR-9 cm			SFM		
**Data**	**PSNR↑**	**SSIM↑**	**LPIPS↓**	**PSNR↑**	**SSIM↑**	**LPIPS↓**	**PSNR↑**	**SSIM↑**	**LPIPS↓**
Office Area 1	**28.09**	**0.870**	**0.282**	26.40	0.850	0.392	24.08	0.846	0.387
Office Area 2	**32.27**	**0.938**	**0.235**	23.12	0.842	0.407	18.03	0.791	0.372
Underground Garage	**29.87**	**0.892**	**0.151**	25.86	0.822	0.393	17.78	0.681	0.485

**Table 7:** Training time summary.

Method	Office Area 1			Office Area 2			Underground Garage		
	**Single**	**Five**	**Titan**	**Single**	**Five**	**Titan**	**Single**	**Five**	**Titan**
Instant- NGP	0.32 h	0.31 h	0.48 h	0.30 h	0.29 h	0.45 h	0.31 h	0.33 h	0.49 h
Nerfacto	0.31 h	0.32 h	0.49 h	0.30 h	0.31 h	0.46 h	0.32 h	0.30 h	0.50 h
Nerfacto- huge	8.02 h	11.98 h	18.11 h	7.15 h	10.45 h	15.75 h	5.01 h	8.24 h	12.98 h
READ	9.12 h	14.34 h	15.88 h	8.05 h	13.09 h	14.99 h	6.07 h	10.12 h	12.34 h
Gaussian Splatting	4.21 h	10.65 h	19.57 h	4.08 h	9.33 h	16.88 h	3.45 h	7.12 h	14.42 h

**Table 8:** Storage consumption summary. The storage unit is mega bytes (MB).

Method	File Type	Office Area 1			Office Area 2			Underground Garage		
		**Single**	**Five**	**Titan**	**Single**	**Five**	**Titan**	**Single**	**Five**	**Titan**
Instant- NGP	.ckpt	189.68	191.7	192.75	189.34	190.09	190.64	185.91	190.89	191.69
Nerfacto	.ckpt	168.41	170.81	172.06	168.01	168.90	169.55	168.21	169.86	170.81
Nerfacto- huge	.ckpt	585.66	587.90	589.15	585.11	585.99	586.64	585.31	585.95	587.91
READ	Net: .pth	115.54	115.54	115.54	115.54	115.54	115.54	115.54	115.54	115.54
	Tex: .pth	91.90	91.90	91.90	81.60	81.60	81.60	139.86	139.86	139.86
Gaussian Splatting	.ply	367.32	557.67	612.54	163.89	180.39	224.54	520.06	538.71	598.38
raw pcd 5 mm interval	.ply	1228.84			1085.44			2181.12		
raw pcd 3 cm interval	.ply	43.0			38.2			65.5		

## Data Availability

The sensor datasets and ground truth data utilized in this work will be available online at http://ivgm-dataset.github.io/ (accessed on 29 August 2024).
